# Systematically Analyzing the Pathogenic Variations for Acute Intermittent Porphyria

**DOI:** 10.3389/fphar.2019.01018

**Published:** 2019-09-13

**Authors:** Yibao Fu, Jinmeng Jia, Lishu Yue, Ruiying Yang, Yongli Guo, Xin Ni, Tieliu Shi

**Affiliations:** ^1^Center for Bioinformatics and Computational Biology, and the Institute of Biomedical Sciences, School of Life Sciences, East China Normal University, Shanghai, China; ^2^Big Data and Engineering Research Center, Beijing Key Laboratory for Pediatric Diseases of Otolaryngology, Head and Neck Surgery, MOE Key Laboratory of Major Diseases in Children, Beijing Children’s Hospital, National Center for Children’s Health, Beijing Pediatric Research Institute, Capital Medical University, Beijing, China; ^3^Biobank for Clinical Data and Samples in Pediatrics, Beijing Children’s Hospital, National Center for Children’s Health, Beijing Pediatric Research Institute, Capital Medical University, Beijing, China; ^4^Department of Otolaryngology, Head and Neck Surgery, Beijing Children’s Hospital, National Center for Children’s Health, Capital Medical University, Beijing, China; ^5^National Center for International Research of Biological Targeting Diagnosis and Therapy, Guangxi Key Laboratory of Biological Targeting Diagnosis and Therapy Research, Collaborative Innovation Center for Targeting Tumor Diagnosis and Therapy, Guangxi Medical University, Nanning, China

**Keywords:** acute intermittent porphyria, *HMBS* gene, genotype and phenotype relationship, hypergeometric test, variation ethnic distribution difference, *PPARA* gene

## Abstract

The rare autosomal dominant disorder acute intermittent porphyria (AIP) is caused by the deficient activity of hydroxymethylbilane synthase (HMBS). The symptoms of AIP are acute neurovisceral attacks which are induced by the dysfunction of heme biosynthesis. To better interpret the underlying mechanism of clinical phenotypes, we collected 117 *HMBS* gene mutations from reported individuals with AIP and evaluated the mutations’ impacts on the corresponding protein structure and function. We found that several mutations with most severe clinical symptoms are located at dipyromethane cofactor (DPM) binding domain of HMBS. Mutations on these residues likely significantly influence the catalytic reaction. To infer new pathogenic mutations, we evaluated the pathogenicity for all the possible missense mutations of *HMBS* gene with different bioinformatic prediction algorithms, and identified 34 mutations with serious pathogenicity and low allele frequency. In addition, we found that gene *PPARA* may also play an important role in the mechanisms of AIP attacks. Our analysis about the distribution frequencies of the 23 variations revealed different distribution patterns among eight ethnic populations, which could help to explain the genetic basis that may contribute to population disparities in AIP prevalence. Our systematic analysis provides a better understanding for this disease and helps for the diagnosis and treatment of AIP.

## Introduction

Acute intermittent porphyria (AIP) is a rare autosomal dominant disorder caused by the deficient activity of hydroxymethylbilane synthase (HMBS), which is also referred as porphobilinogen deaminase (PBGD), the third enzyme in the pathway of heme biosynthetic (Gill et al., 2009; Chen et al., 2018). Clinically, most of the symptomatic AIP patients are women (Pulgar et al., 2019), especially in their reproductive age (Innala et al., 2010). It is commonly recognized that sex steroids play an important role for the clinical manifestations in porphyria in women and they act as inducers in heme biosynthesis (Innala et al., 2010). The clinical symptoms of AIP patients include acute recurrent abdominal pain, and often are accompanied by gastrointestinal disorders such as nausea, vomiting and constipation (Yang et al., 2015; Yang et al., 2016; Duque-Serrano et al., 2018). In addition, hypertension, tachycardia, hyponatremia and motor weakness are often present (Puy et al., 2010; Kong et al., 2013). Mental changes include anxiety, depression, agitation, etc. (To-Figueras et al., 2006; Duque-Serrano et al., 2018). Most of the clinical features of an attack are caused by the effects on the nervous system and the characteristics of the symptoms are not so obvious (Stein et al., 2017). This is one of the reasons why it can easily be misdiagnosed. In many cases, patients suffer from frequent convulsions and seizures. But patients with severe psychiatric symptoms such as psychosis, hallucinations and delirium are rare. Due to the similarity to other disease symptoms, it is a challenge for clinicians to diagnose patients with intermittent porphyria at their first attack. Porphyrin levels in urine and blood is a useful diagnostic index for patients with a suspected acute attack of AIP.

Heme is mostly synthesized in erythropoietic cells and liver cells, and plays an essential role in the synthesis of hemoproteins such as hemoglobin, cytochromes, myoglobin, catalase, and peroxidase, all of which are important for the transportation of oxygen and the oxidation–reduction reactions (Karim et al., 2015). HMBS is the third enzyme in the heme biosynthesis pathway, in which the synthesis of ALA (5-aminolaevulinic acid) is one of the most important controlling steps for heme formation. ALAS (5-aminolaevulinic acid synthase) is the enzyme that controls the first step in the heme biosynthesis and encoded by two different genes: *ALAS1* (ubiquitously expressed in every human cell) and *ALAS2* (only expressed in erythroid) (Puy et al., 2010). ALAS1 acts as the rate limiting enzyme in the heme synthesis pathway in the liver and can be controlled by heme through negative feedback regulation loop, as heme down-regulates the transcription of ALAS1 (Ajioka et al., 2006). The partial deficiency of HMBS activity can hinder the heme synthesis and the negative regulation mechanism could lead to the excessive accumulation of heme precursors such as ALA and PBG in tissues, which may trigger acute attacks (Tracy and Dyck, 2014).

**Figure 1:**
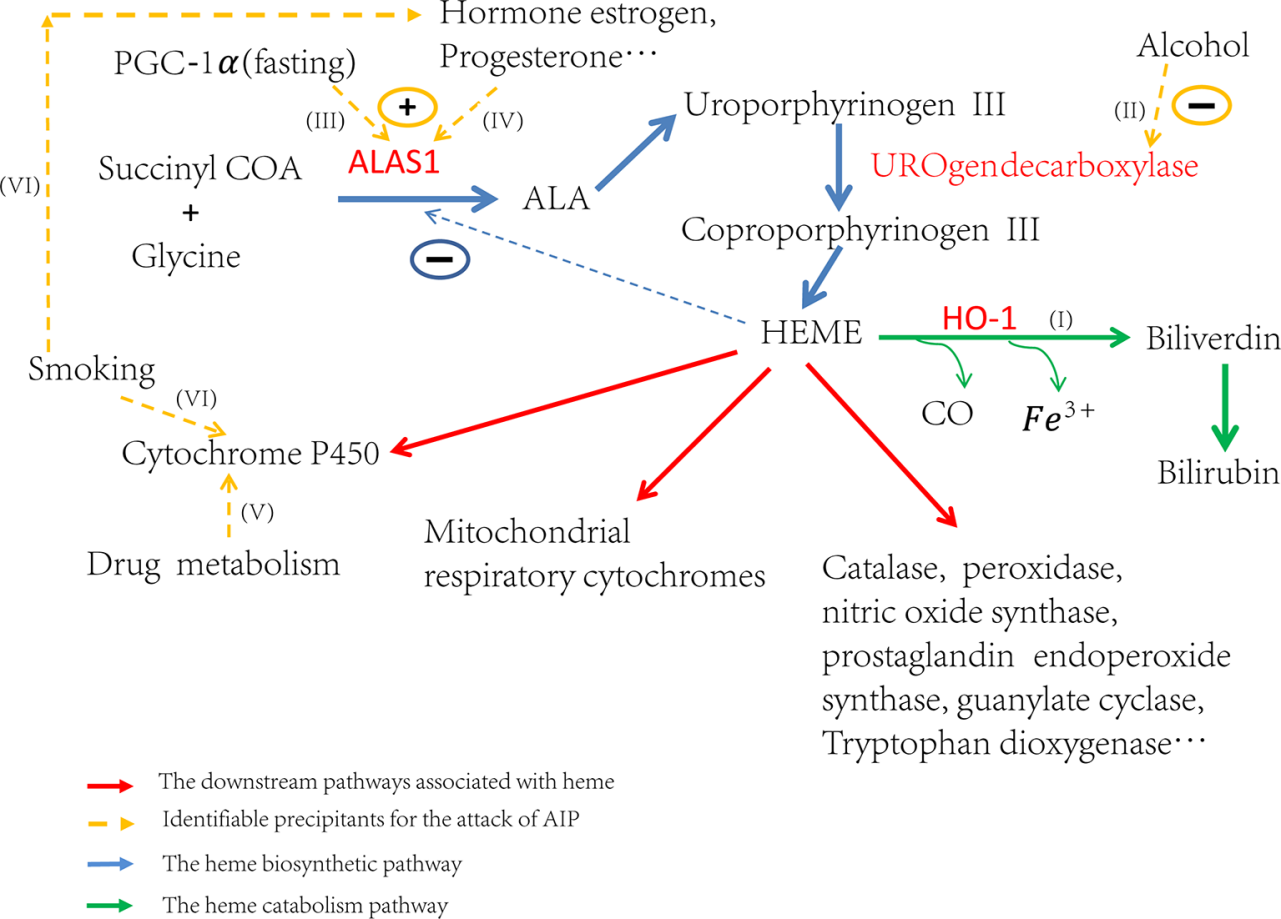
Trigger factors’ effect on the regulation of heme biosynthesis. If **(I)** Induction of HO-1 can catabolize heme and then ALAS1 is de-repressed through the negative regulation network. If **(II)** Alcohol can induce attacks through the inhibition of uroporphyrinogen decarboxylase and subsequently lead to the accumulation of porphyrin precursors. If **(III)** Fasting can upregulate the transcription of ALAS1 through the increased production of PGC-1α. If **(IV)** The hormones estrogen, progesterone and testosterone can increase the enzyme activity of ALA synthase. If **(V)** The drug metabolism can stimulate heme production, particularly those which need metabolism throughout the cytochrome P450 system, resulting in the excess production of heme precursors. If **(VI)** The elements of cigarette smoke may stimulate the P450 system and the steroid hormones balance in smokers may be altered.

Attacks of AIP can be induced by many different factors. These factors contribute to the attacks by inducing *ALAS1* transcription or the activity of ALA synthase, either directly or indirectly (**Figure 1I**) (Stein et al., 2017). Some triggers increase the demand for heme in the liver which de-represses the transcription of *ALAS1* thereafter (Ajioka et al., 2006). Others, like hormones estrogen and progesterone, increase the activity of ALA synthase (**Figure 1IV**), which partially explains why AIP often attacks during the luteal phase of the menstrual cycle (Junior et al., 2017). Up to now, the identified factors that induce the attacks include alcohol, smoking, nutritional factors, hormonal factors, and the usage of drugs etc. (**Figures 1II, VI, III, V**) (Handschin et al., 2005; Windebank et al., 2005; Tracy and Dyck, 2014; Karim et al., 2015). All of these factors affect the heme production pathway, and then lead to the over-accumulation of toxic heme precursors (**Figure 1**).

The mechanism of psychiatric symptoms in AIP is not fully understood. The current researches suggest that the accumulation of ALA is the prime culprit in the damage to nervous system. One of the hypotheses is the structural similarity between ALA and γ-aminobutyric acid (GABA), the accumulation of ALA may affect normal GABA function in the nervous system (Windebank and Mcdonald, 2005; Tracy and Dyck, 2014). The excess of ALA leads to the pronounced HO (heme oxygenase) activity, resulting in deregulation of the cholinergic system, increasing oxidative stress, affecting activity and expression of NOS (nitric oxide synthases) (Lavandera et al., 2016), decreasing GABAergic neurons’ activity (Brennan and Cantrill, 1979) and increasing glutamate release (Satoh et al., 2008; Duque-Serrano et al., 2018).

With the rapid development of next generation sequencing (NGS) technology (Gill et al., 2009; Shen, 2018), more and more disease related mutations have been detected. Up to now, the Human Gene Mutation Database (HGMD) (http://www.hgmd.cf.ac.uk/ac/) has collected a total of 421 different mutations on gene *HMBS*, including missense/nonsense, splicing, small deletions, small insertions, small indels, and gross deletions, etc. Patients with different mutations present various severity of clinical symptoms. However, many mutations’ effect on the protein structure and their relationship with phenotype are not fully explored. For example, there are cases that patients’ family members with the same mutation are asymptomatic (Li et al., 2015). Also, patients with the same mutation exhibit clinical manifestation of various severity. Therefore, to obtain a comprehensive landscape of the effects of different mutations, we conducted analysis on the effects of mutations based on the second structure and the three-dimensional structure of corresponding proteins and tried to infer the relationships between phenotypes and genotypes. Considering the complexity of metabolic processes in human body, we supposed that other genes which interact with genes or proteins in the heme synthesis pathway may also play roles in the attack of AIP. To identify new genes that potentially contribute to this disease, we conducted both protein–protein interaction network and pathway enrichment analyses. In addition, we also explored the genetic difference among different ethnic groups based on reported patients’ cases for this disease. We analyzed the risk allelic frequencies among eight different populations world-wide. Our research results provide overview picture for the distribution of AIP mutations and shed light on better understanding of AIP disease.

## Materials and Methods

### Analysis of the Relationship Between Phenotypes and Genotypes

We collected the clinical symptoms and corresponding mutation information of 117 patients from related resources and literature (**Table S1**). All of those clinical and genetic data have been inputted into eRAM and PedAM systems (Jia et al., 2018a; Jia et al., 2018b). Based on the different clinical symptoms, we classified patients into three categories: severe, moderate, and mild. Our classification criteria include frequency of attacks, blood pressure, heart rate, mental condition, blood tests result, serum sodium concentration, the extent of abdominal pain, vomiting, and nausea, etc. (Zanella et al., 2007). Then we analyzed the corresponding missense mutations in each phenotype class.

In order to understand the effect of these missense mutations on HMBS protein structure, we mapped those mutations to the crystal structure (PDB: 5M7F, Resolution: 2.78-*Å*) (Pluta et al., 2018) to study how the mutations affect the structure of the protein and then evaluated the relationship between mutations and clinical symptoms. For the visualization of the three-dimensional structure of protein, we used a web tool (http://www.sbg.bio.ic.ac.uk/∼ezmol/). We also studied the interactions between different amino acid residues using the Residue Interaction Network Generator (RING) (http://protein.bio.unipd.it/ring/) and interpreted the interrelation between different residues, including 5M7F (Human porphobilinogen deaminase in complex with DPM cofactor) and 5M6R (Human porphobilinogen deaminase in complex with reaction intermediate).

To further explore the mutation’ effect on the HMBS protein structure, we performed multi-sequence alignment for those homologous protein sequences of different species extracted from the UniGene database (https://www.ncbi.nlm.nih.gov/unigene/?term=HMBS) with bioEdit software and inspected the conservation of amino acids (Mahdavi et al., 2018).

**Figure 2:**
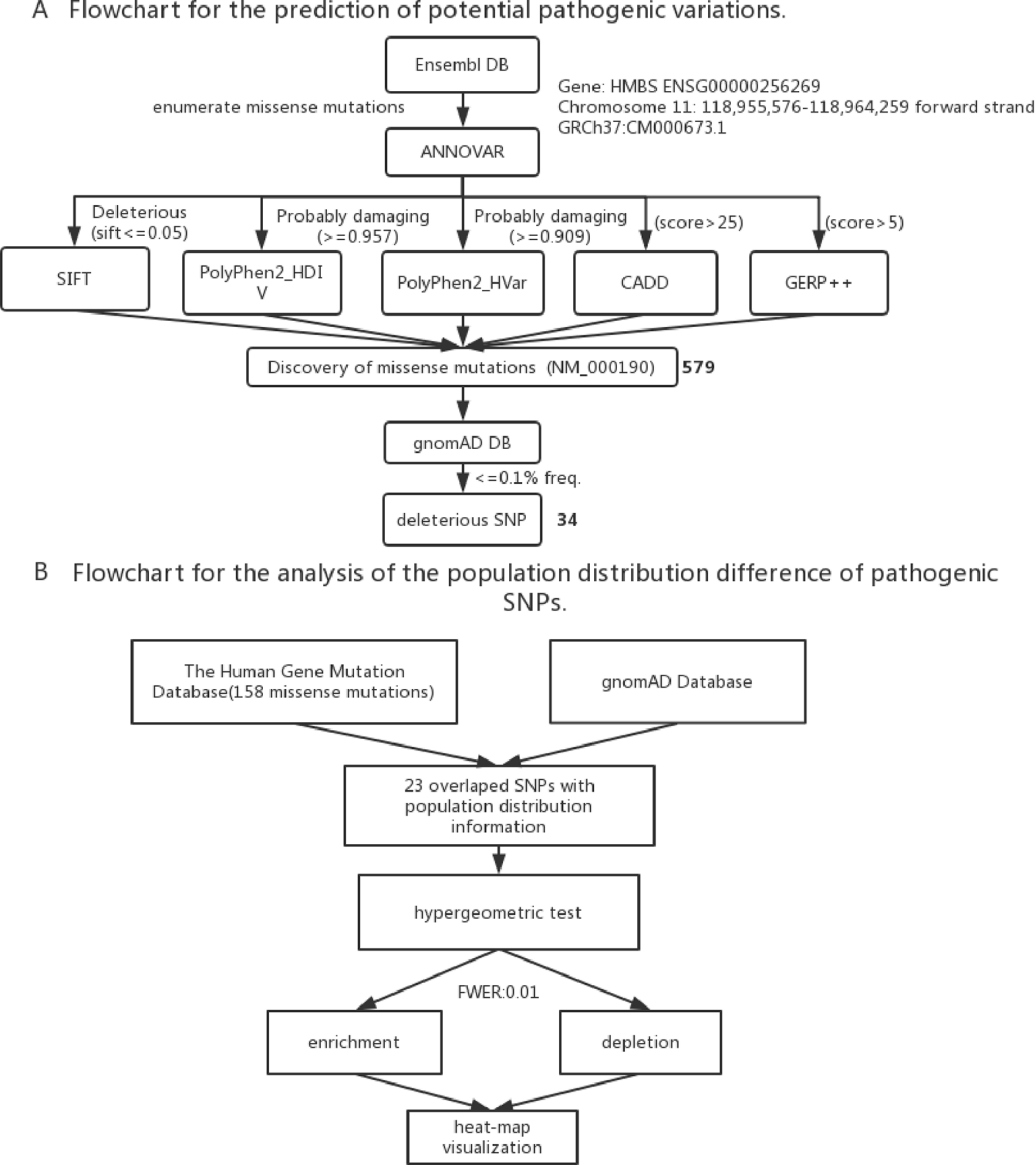
**(A)** Flowchart for the prediction of potential pathogenic SNPs. **(B)** Flowchart for the analysis of the population distribution difference of pathogenic SNPs.

To identify the new pathogenic variations, we obtained the FASTA file of gene *HMBS* from Ensembl database (GRCh37:CM000673.1), and then listed all the missense variations with the method of enumeration (**Table S2**). The analysis of the pathogenic effects of these missense variations on *HMBS* gene was conducted by using five prediction algorithms: SIFT, PolyPhen 2 HDIV, PolyPhen 2 Hvar, CADD, GERP ++ (**Table 1**). At first, 579 missense variations which met the selection criteria were identified. The cutoff values for selecting the possible pathogenic mutations were set larger than the average of predicted score of collected mutations to ensure the reliability of prediction results. Next, we selected those missense variations with frequency less than 0.1% based on the gnomAD database (http://gnomad.broadinstitute.org/) and obtained 34 deleterious variations (**Figure 2A**) (**Table S3**).

**Table 1:** Criteria for selecting predicted Pathogenic SNPs.

Protein prediction algorithm	Cutoff-Value	Interpretation
SIFT	D	D:Delterious(sift< = 0.05)
PolyPhen 2 HDIV	D	D:Probably damaging(> = 0.957)
PolyPhen 2 Hvar	D	D:Probably damaging(> = 0.909)
CADD	Score >25	10 means 10% percentile highest scores; 20 means 1% percentile highest scores; 30 means 0.1% percentile highest scores
GERP++	Score >5	High scores are more deleterious

### Prediction of New Associated Genes

As the control step of heme biosynthesis pathway, ALAS1 plays a rate-limiting role which is regulated by heme through the negative feedback loop. So we tried to predict new AIP-related pathogenic genes from the perspective of interactions between ALAS1 and other genes. STRING (https://string-db.org/cgi/input.pl) is a database for known and predicted protein-protein interactions and presents the physical and functional associations between proteins. inBio Map™ (https://www.intomics.com/inbio/map.html) is another protein-protein interaction platform known for its high coverage and high quality (Li et al., 2017). We used the two databases to identify the possible interacting genes for *ALAS1*.

To validate our results and explore the underlying mechanisms of those predicted genes, we conducted the protein functional analysis and pathway enrichment analysis using Reactome database (https://www.reactome.org), which is a relational database of signaling and metabolic molecules (Fabregat et al., 2018).

To check the gene expression pattern of those interacting proteins, we used the Genotype-Tissue Expression (GTEx) database (https://gtexportal.org/home/). Furthermore, to validate the predictive associated gene, we studied their protein or gene function based on UniProt database (https://www.uniprot.org/) and published literature.

### Distribution Difference of Variations Among Populations

To study the population distribution difference of AIP — associated variations in allele frequency, we used the published 158 missense mutations in *HMBS* gene from HGMD and obtained their population distribution information from gnomAD database, the ethnic groups include South Asian, European (Non-Finnish), African, East Asian, Ashkenazi Jewish, European (Finnish), Latino and other. In the end, there were 23 variations with population distribution information available (**Table S4**).

To assess if the risk allele of an AIP — associated variations is enriched or depleted (separated) significantly in each of the 8 populations, we performed 16 (2 × 8) hypergeometric tests for each variation (Mao et al., 2017). If the p-value of enrichment is less than the p-value of depletion, then it is over-represented, but may not significantly over-represented. If the p-value of enrichment is greater than the p-value of depletion, then it is depleted. To control a family-wise error rate (FWER) of 0.01, we used a raw p-value of 0.01/368 = 2.717E-5 as a cutoff.

To visualize variation enrichment/depletion patterns in 8 populations, we first transformed the hypergeometric testing p-values by *log*_10_. After that, we used Seaborn package of Python to generate hierarchical clustering heat-map based on enrichment/depletion p-values (*log*_10_ based) of risk variations in different populations (**Figure 2B**). If an AIP-associated variation is enriched in a population, we used the negative of *log*_10_ of the enrichment p-value to represent the variation in the cluster heat-map. In contrast, if a variation is depleted in a population, the value of *log*_10_ of the depletion p-value was used.

## Results

### The Relationship Between Genotypes and Phenotypes

**Figure 3:**
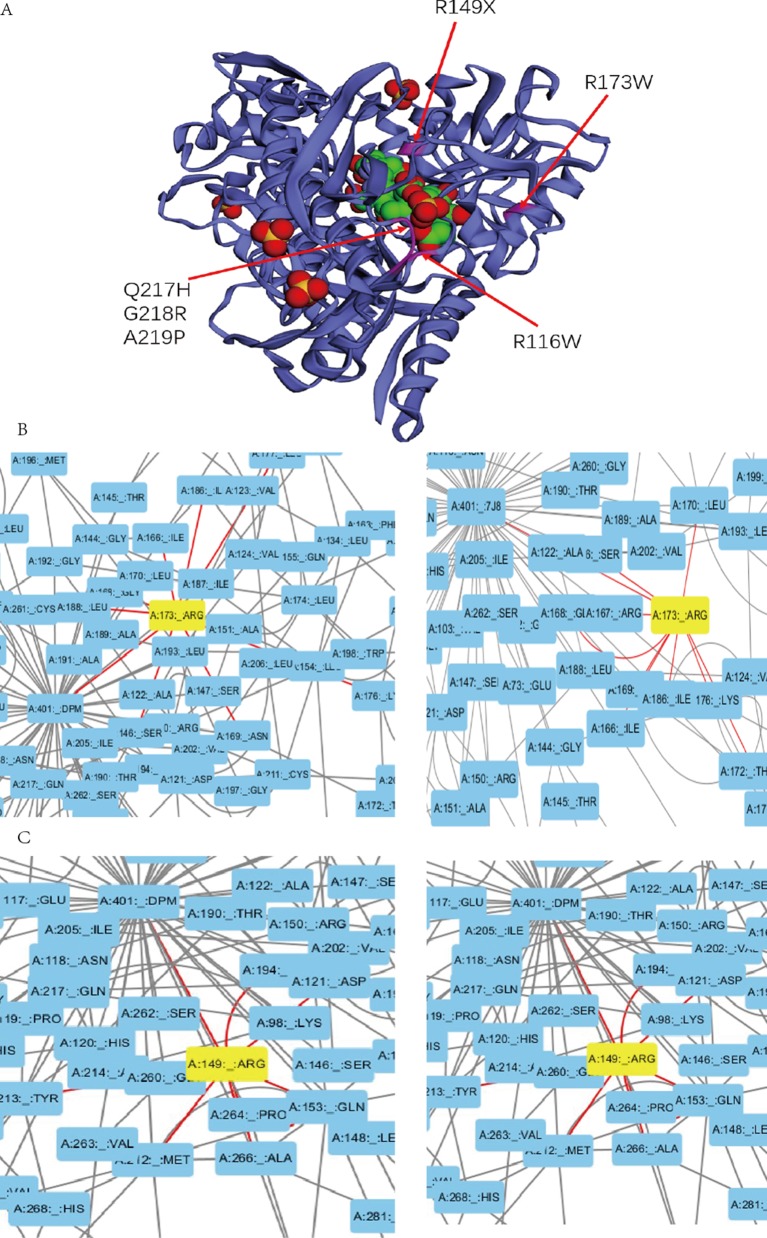
**(A)** Mutations with severe clinical symptoms are near the binding site for DPM (dipyrromethane) cofactor at active site on the crystal structure of human HMBS enzyme (5M7F). **(B)** **(C)** Residue A173 and R149 interacts with the DPM cofactor (5M7F) and reaction intermediate (5M6R) at atomic level in the HMBS protein structure by the Residue Interaction Network Generator (RING) software.

Previous studies experimentally characterized two novel mutations by comparing them with wild-type (wt) *HMBS* (Bustad et al., 2013). Our collected clinical data show that patients with mutation R116W and R173W have severe clinical symptoms, which is consistent with previous study that R116W mutation leads to the protein defection in conformational stability while R173W impacts both enzyme kinetics and conformational stability (Bustad et al., 2013). In the residue interaction network of 5M6R and 5M7F, R173 interacts with many other residues, including S146, I166, G168, N169, K176, L177, I186, and L188. Besides, it also interacts with the reaction intermediate (*7J8*) and dipyrromethane (DPM) cofactor (**Figure 3B**). R173 locates at α-helices structure in domain 2 (**Figure 3A**), mutations on this residue will generate severe defection to the catalytic proteins, which is very similar to the effect of mutation R116W. In addition, residue R116 locates at the hinge-bending region of domains 1 and 2, mutations on it are believed to affect the binding ability of its substrate.

In addition, mutations R149X, Q217H, G218R, A219P and A330P can also lead to severe clinical manifestations. In the residue interaction network, residues R149, Q217, G218 and A219 all have direct interactions with both the reaction intermediate and DPM cofactor (**Figures 3C**, **S1**). Residue A330 locates at α-helices structure in domain 3. Although A330 has no direct interaction with the reaction intermediate, the nonsynonymous mutations on it could disrupt the secondary structure of protein and affect other five residues. Mapping the missense mutation to the crystal structure of human HMBS enzyme showed that residues Q217, G218, and A219 are all in close proximity to the attachment site of dipyrromethane (DPM) cofactor (**Figure 3A**). The substitution of these residues may affect the cofactor’s binding which impact the catalytic reaction. Multiple sequence alignment revealed that all these four residues are strongly conserved among many vertebrate species (**Figure S2**). Taken together, it is reasonable to hypothesize that missense mutations on these residues can lead to severe clinical manifestations.

The corresponding clinical symptoms for mutations R26C, D99N, R167W, G168X, Q194X, and G221D are relatively moderate. Although they interact with the reaction intermediate and DPM cofactor directly in the residue interactive network, their locations on the protein 3D structure reveal that they are relatively away from the active site (**Figure S3**), and only a few other residues interact with these residues. Therefore, mutations on these residues most likely lead to relatively moderate defection compared to the normal activity of HMBS. Patients with mutations T35M, G111R, and L238P present a mild clinical phenotype. Those amino acids locate far from the active site based on the three-dimensional structure and have no direct interaction with the reaction intermediate and DPM cofactor. Besides, they have small number of interacting residues in the residues interacting network. Mutations on these residues may have less effect on the protein structure and these mutations should have relatively low pathogenic impact, which can partially explain why patients carrying those mutations only present mild clinical symptoms.

To identify other potential pathogenic mutations, we conducted the prediction for potential deleterious variations (**Figure 2A**). First, according to the cutoff-value of five different bioinformatics algorithms, 579 missense mutations were selected as the most deleterious mutations. Then we checked the allele frequencies of all the selected mutations in gnomAD database and excluded those mutations whose frequency are more than 0.1%. As the result, we obtained 34 missense mutations as the potential pathogenic variations. Residues R22, R26, M56, S75, E80, E82, E86, N88, E89, L97, R116, A122, S141, V142, V143, T145, A152, P159, R167, T172, D178, A189, A219, G260, V265, V282, G287, G317, A331, and G346 may have potentially important roles for the normal activity of HMBS. Remarkably, mutations on residues R26, R116, R167, and R219 have been reported to be pathogenic and present relatively severe clinical manifestations, which provide the evidence to support our prediction results.

In addition, taking residues L97, A122, A145, A189, G260, and V265 as examples, the RING analysis result showed that all these five mutations had connections with DPM cofactor (5M7F) and reaction intermediate (5M6R). Those mutations could affect the normal binding of catalytic cofactor and reaction intermediate, which result in the abnormality of the reaction. Overall, these new identified variations could be the candidates that contribute to the disease.

### Prediction for Pathogenic Candidate Genes

There are 10 possible interactive partners for ALAS1 protein from STRING database and 16 from inBio Map™ database. Based on the protein functional analysis, we noticed that gene *PPARA* (peroxisome proliferator-activated receptor alpha) had obvious function overlap with ALAS1.

Heme is an iron porphyrin compound which plays as the prosthetic group of hemoglobin, myoglobin, cytochrome, peroxidase, and catalase. Most drug metabolisms are closely associated with the cytochrome P450 enzymes, so the drugs, particularly those ones metabolized through the cytochrome P450 system, can increase hepatic heme turnover (**Figure 1V**) and affect the ALAS1 through the negative feedback regulation loop, thus leading to the excess production of heme precursors. Protein functional analysis results showed that gene *PPARA* had a direct regulation of the transcription of cytochrome P450 gene *CYP2C8* (Thomas et al., 2015) and hepatic cytochrome P450 3A4 (*CYP3A4*) (Thomas et al., 2013). Therefore, mutations on *PPARA* can affect the cytochrome P450 system, and consequently may have an influence on the heme biosynthesis pathway indirectly, resulting in acute of intermittent porphyria.

Pathway enrichment analysis with Reactome showed that *ALAS1* shared three same pathways with *PPARA*, they are metabolism of lipids and lipoproteins, fatty acid triacylglycerol and ketone body metabolism and *PPARA* activates gene expression respectively. One of the clinical symptoms of AIP is hyperlipidemia (HP: 0003077), which is closely associated with the abnormal metabolism of lipids. And it is well known that the activation of the PPARα can reduces hyperlipidemia (Kimura et al., 2013). The relationships between the phenotypes of hyperlipidemia and the regulation mechanism of lipid metabolism indicates that gene *PPARA* is likely to be associated with AIP.

**Figure 4:**
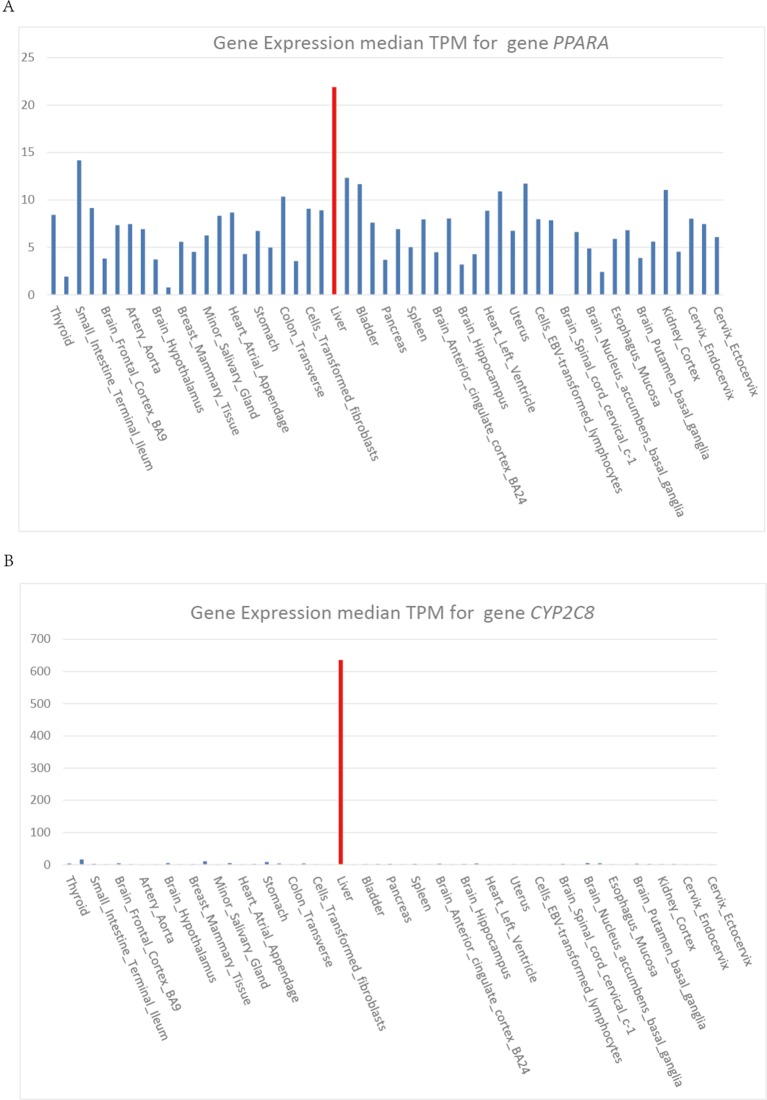
**(A)** **(B)** Gene expression median TPM for gene *PPARA* and *CYB2C8*.

Heme is synthesized mainly in erythropoietic cells (80%) and liver parenchymal cells (15%). ALAS1 acts as the rate limiting enzyme in the production of heme in liver and can be regulated by heme. Whereas, in erythroid cells, ALAS2 acts as the catalyzing enzyme in the first step of heme biosynthesis and the synthetic rate is limited by iron availability (Puy et al., 2010). The cytochrome P450 gene *CYP2C8* and *CYP3A4* are highly expressed in liver, which show the same expression pattern as gene *PPARA* (**Figure 4**). Gene *PPARA* can affect the drug metabolism by the direction regulation of transcription of *CYP2C8* and *CYP3A4*, and thus affect the heme biosynthesis pathway. These functional associations between *PPARA* and *ALAS1* suggest that gene *PPARA* may play an important role in the mechanisms of AIP attacks and could act as the possible pathogenic gene.

### The Distribution Difference of Variations Among Populations

**Figure 5:**
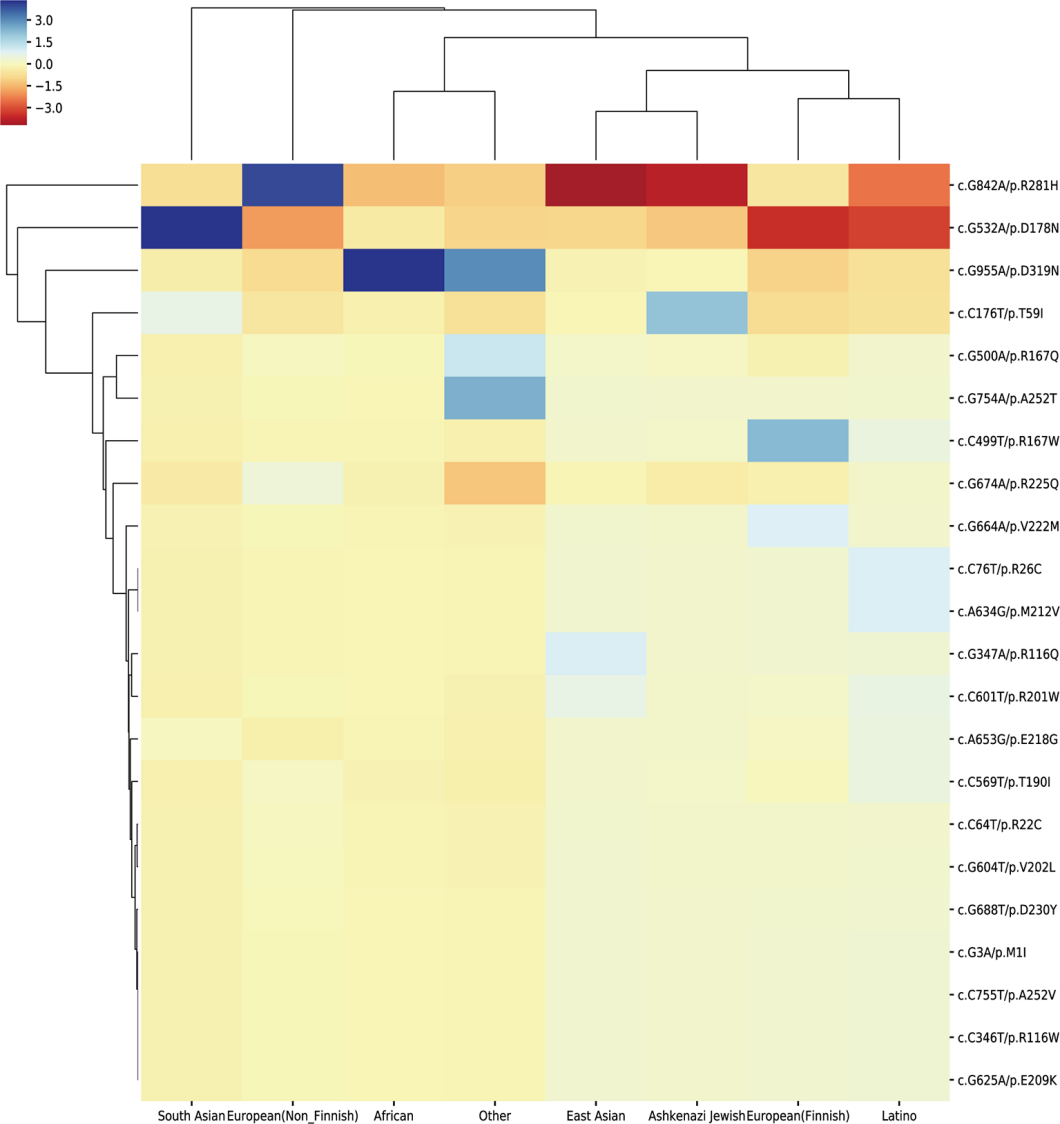
Cluster heat-map shows the significance of the 23 pathogenic variations enriched or depleted in each population compared with the worldwide average. Each row represents a variation, and each column represents a population. A unit in the cluster heat map is colored based on the *log*_10_ of p-value.

We extracted the variation frequency of 23 *HMBS* gene mutations among eight different ethnic groups from gnomAD and assessed if a given variation was significantly enriched or depleted in different ethnic groups by hypergeometric test. The heat-map (**Figure 5**) demonstrated that missense mutation R167W was enriched in Finnish people, D319N was significantly enriched in African and depleted in the rest of regions. Mutation D178N was enriched and R281H was depleted in South Asian compared with the global average, whereas the distribution of the two variations was in the opposite situation in Non-Finnish European. Besides, East Asian, Ashkenazi Jewish, European (Finnish) and Latino exhibit similar allele enrichment/depletion patterns and South Asian, Non-Finnish European and African share overall similar distribution patterns for the 23 mutations.

The distribution frequency of the 23 variations reveals their different patterns among 8 populations. The possible explanation for these results is that the allele abundance may be shaped by different environmental factors and evolution patterns. Because of the small amount of variations used in our analysis, the distribution pattern may not be so obvious. More pathogenic variations could provide better view for the pattern difference among populations.

## Discussion

In this study, we have explored the triggers and their mechanisms for acute attacks in AIP. Currently, many attacks have no clearly identifiable triggers. For those suspected patients, close attention should be paid to avoid these precipitants in their daily life. Although the integral and exact mechanisms of nervous system dysfunction are not fully understood, current works have offered basic guidelines to analyze the pathogenesis of variants and provided useful information for the diagnosis and the treatment of the disease.

Based on the collected mutation pattern and clinical information, we analyzed the relationship between genotypes and phenotypes. Previous research has made the conformational stability and activity analysis of several HMBS mutations, they interpreted the molecular mechanisms of HMBS mutations and inferred the connection between genotypes and phenotypes to certain extent (Bustad et al., 2013). By mapping the mutations to the protein crystal structure and conducting the residues interaction analysis, we have obtained more comprehensive view about how the mutations affect the protein function. Further experiment of molecular biology can verify our interpretation about the specific influence of those mutations on the protein structure. We also used different bioinformatics prediction algorithms to evaluate the pathogenicity of enumerated possible missense mutations. Some of the new predicted deleterious mutations may affect the binding site for the dipyrromethane cofactor at the active site, which provides new resource to better understand the mutation spectra of the disease.

By protein functional analysis and pathway enrichment analysis, we found that gene *PPARA* was correlated with the attacks of AIP through the direct regulation of the transcription of cytochrome P450 gene *CYP2C8*, which may affect the turnover of heme in the liver. Besides, in the *PPARA* activating gene expression pathway, we also noticed that *NRF1* and *PPARGC1B* (PGC-1β) could regulate the expression of *ALAS1*. Experiment on mouse has demonstrated that PGC-1β (*PPARGC1B*) binds NRF1 and co-activates genes regulated by NRF1 (Ivanova et al., 2013). The dysfunction of *NRF1* and *PPARGC1B* may be associated with this disease. Also, gene *ESRRA* can affect the normal expression of *ALAS1*, indicating that the normal heme biosynthesis pathway may be affected and gene *ESRRA* may be the potential new pathogenic gene which is related to the attacks of AIP.

However, one limitation of our study is that we have only focused on those mutations located around the binding region for DPM at the active site. Other mutations located away from the region could also play an important role in the three-dimensional structure of protein and affect the activity of enzyme. Another limitation of our work is that we only have studied the missense mutations in the coding region of *HMBS* gene. We do not consider other mutation types (frameshift, deletion, insertion and nonsense) and mutations in the non-coding region. In addition, larger amount of variations with population distribution frequency should be collected to have a better view for the allelic distribution difference patterns. Nevertheless, our systematic analysis provides a better understanding for this disease and help for the diagnosis and treatment of AIP.

In the past few decades, the diagnosis and therapy of acute porphyria have been paid a lot attention by researchers and clinicians around the world (Jia and Shi, 2017). European Union Directorate-General (EU DG) for Health & Consumers have established an effective network (European Porphyria Network) of specialist porphyria centers throughout the EU. Currently, EPNET (European Porphyria Network, http://porphyria.eu/en/content/acute-porphyria) consists of 33 EU specialist centers from 21 European and candidate countries. They also have associate members from Australia, Brazil, New Zealand, South Africa, and the USA. They collected information for people with AIP, variegate porphyria or hereditary coproporphyria, and their families, which is really helpful for the diagnosis and treatment of patients. However, for a long time, public health agencies and the whole society have paid less attention to rare diseases in China (Li et al., 2017;Ni and Shi, 2017). So, the systematic research on acute porphyria is progressing slowly. To effectively diagnose and treat AIP disease, a systematic collection of patients’ information is urgently needed. Our study should provide more useful information to the study of AIP.

## Author Contributions

In this study, TS, YG and XN designed the study. YF, LY, RY, and JJ conducted the data collection and data analysis. YF, JJ, TS, YG and XN interpreted data. YF and JJ drafted the manuscript. TS, XN, and YG revised and finalized the manuscript. All authors read and approved the final manuscript.

## Funding

This work was supported by the China Human Proteome Project (Grant No.2014DFB30010, 2014DFB30030), National High Technology Research and Development Program of China (863 project) (2015AA020108), National Natural Science Foundation of China (31671377, 81472369 and 81502144), Clinical Application Research Funds of Capital Beijing (Z171100001017051), Beihang University & Capital Medical University Advanced Innovation Center for Big Data-Based Precision Medicine Plan (BHME- 201801) and Shanghai 111 Project (B14019).

## Conflict of Interest Statement

The authors declare that the research was conducted in the absence of any commercial or financial relationships that could be construed as a potential conflict of interest. 

The handling editor is currently editing co-organizing a Research Topic with one of the authors TS, and confirms the absence of any other collaboration.

## Supplementary Material

Figure S1 **(A) (B) (C)** Residues’ interaction with DPM cofactor and reaction intermediate in the residue interaction network.

Figure S2 Multiple sequence alignment revealed that mutation residues are strongly conserved among most vertebrate species.

Figure S3 The locations of residues R26, D99, R167, G168, Q194 and G221 on the crystal structure of human HMBS enzyme (5M7F).

Table S1 One hundred seventeen collected AIP patients’ clinical symptoms and mutation information.

Table S2 All the enumerated missense mutations and their pathogenic evaluation.

Table S3 Thirty four new potential deleterious mutations identified by our selection.

Table S4 Twenty three deleterious mutations with population distribution frequency.
