# Supplementary figures and images for: Systematically Analyzing the Pathogenic Variations for Acute Intermittent Porphyria
Source: Front Pharmacol. 2019 Sep 13;10:1018. doi: 10.3389/fphar.2019.01018 (PMC6753391; doi:10.3389/fphar.2019.01018)

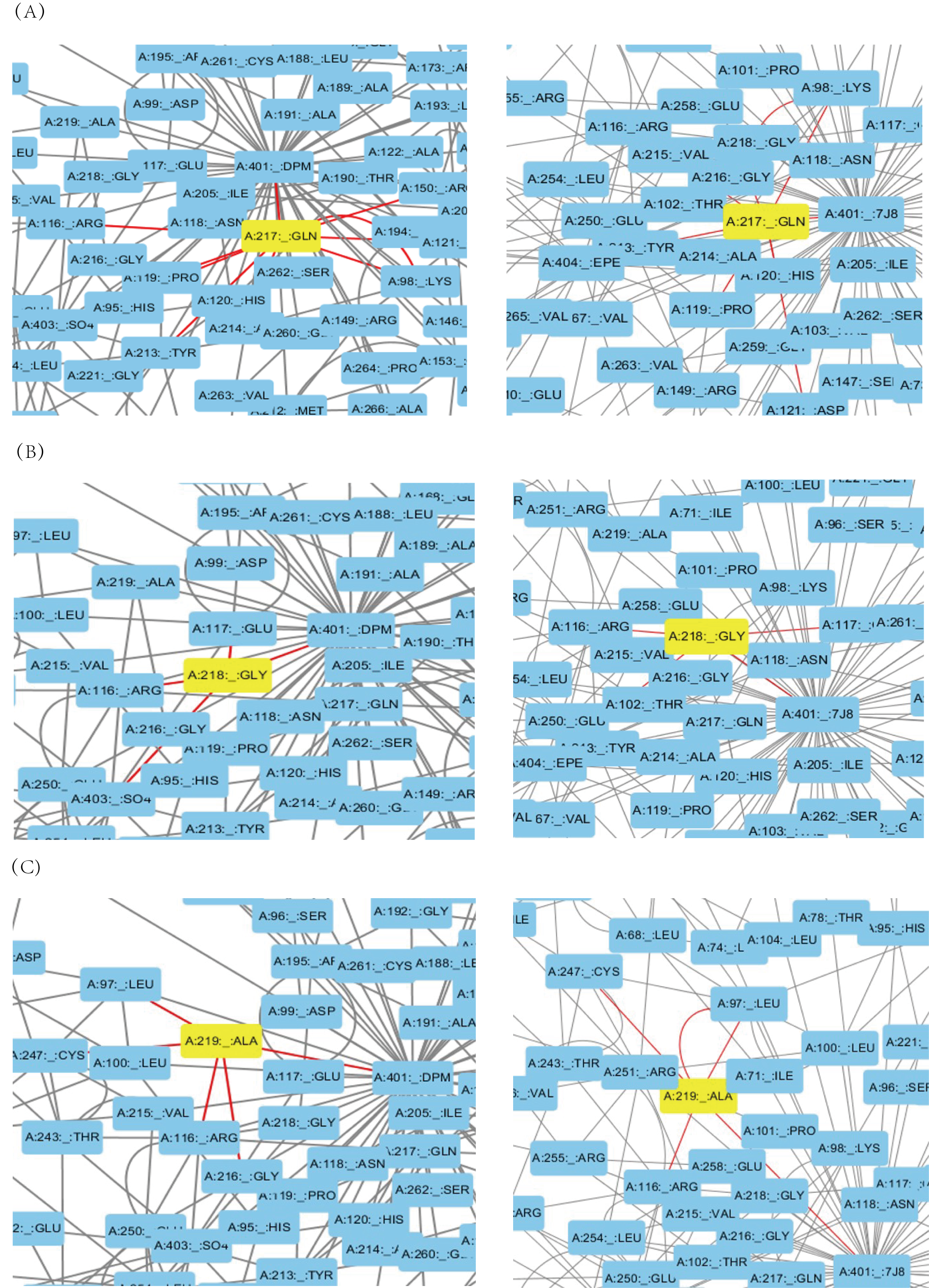

Supplement: Figure S1 — (A) (B) (C) Residues’ interaction with DPM cofactor and reaction intermediate in the residue interaction network. [file Image_1.tif]

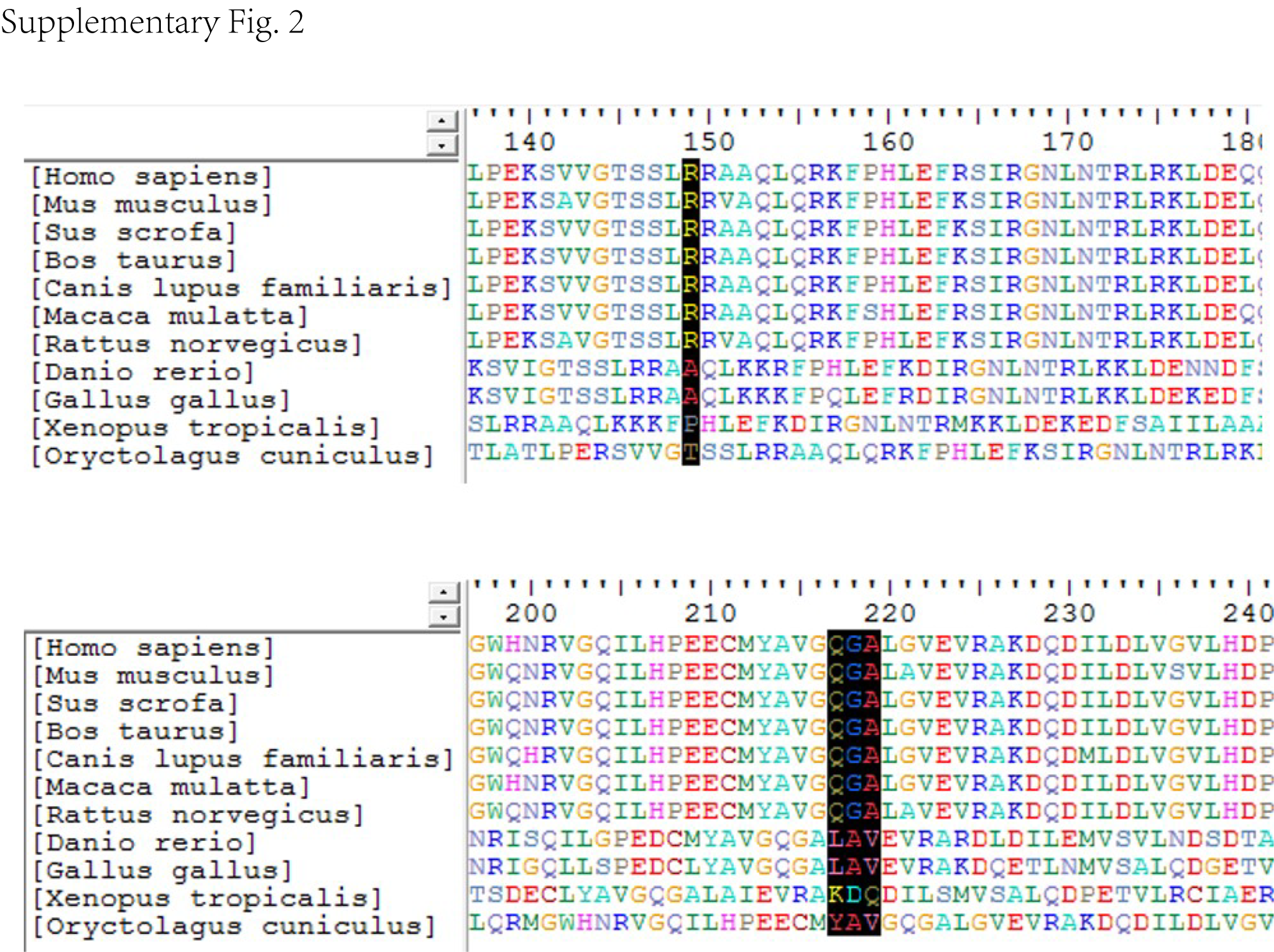

Supplement: Figure S2 — Multiple sequence alignment revealed that mutation residues are strongly conserved among most vertebrate species. [file Image_2.tif]

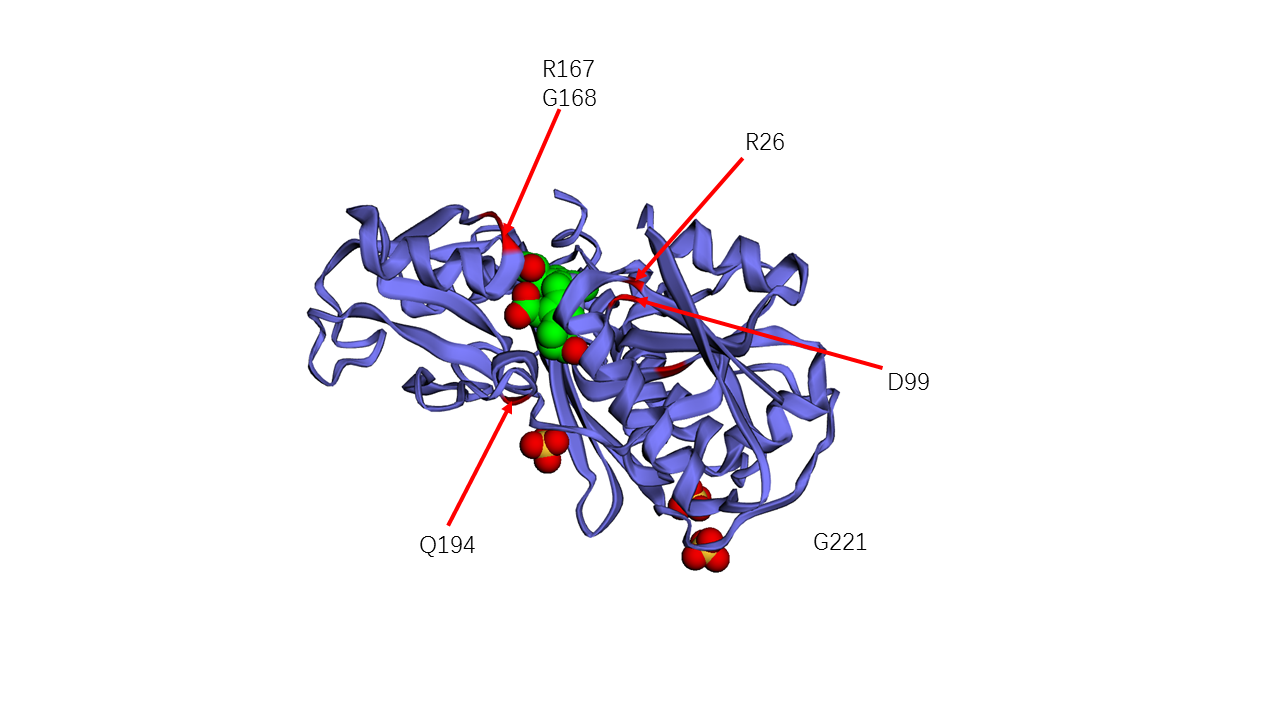

Supplement: Figure S3 — The locations of residues R26, D99, R167, G168, Q194 and G221 on the crystal structure of human HMBS enzyme (5M7F). [file Image_3.tif]
